# Epilepsy control during an epidemic: emerging approaches and a new management framework

**DOI:** 10.1186/s42494-020-00015-z

**Published:** 2020-05-12

**Authors:** Deng Chen, Lina Zhu, Xin Lin, Zhen Hong, Shichuo Li, Ling Liu, Dong Zhou

**Affiliations:** 1 Department of Neurology, West China Hospital, Sichuan University, Chengdu, 610041 China; 2 Department of Neurology, Shanghai Huashan Hospital, Fudan University, Shanghai, 200040 China; 3 China Association Against Epilepsy (CAAE), Beijing, 10044 China

**Keywords:** Epidemic, Epilepsy, Disease control

## Abstract

Epidemics are a big threat to world health. The ongoing pandemic of corona virus disease 2019 (COVID-19) has caused a series of challenges to public health. One such challenge is the management of chronic diseases such as epilepsy during an epidemic event. Studies on this topic are rather limited and the related medical practice is full of uncertainty. Here we review recent development of potential approaches for epilepsy control during an epidemic and propose a new three-level management framework to address these challenges.

## Introduction

Epidemics were and still are a dreadful threat to public health. Despite continuous efforts by scientists, health providers and even the whole society, the incidence of infectious diseases is still rising [1]. In 2019 alone, the WHO has documented over 100 disease outbreaks affecting more than 20 countries [2]. Although most of these outbreaks occurred in resource-limited regions such as Africa, Middle East and South America, other countries are not excluded from the risk. The outbreak of corona virus disease 2019 (COVID-19) beginning in December 2019 is the most recent ongoing pandemic, which has affected millions of people worldwide [3]. By April 10th, 2020, this disease has affected over 1.6 million people and caused 100 000 deaths worldwide.

Epidemics, even not reaching a pandemic level, have dramatic impacts on society that are far beyond disease itself. During the 2003 Severe Acute Respiratory Syndrome (SARS) outbreak in Beijing, international transportation, cargo exchange and tourism were ruined by the epidemic, leading to an estimated loss of 1.4 bn USD, 300 times higher than the cost for controlling the disease itself [4]. Besides economic loss, the crisis in management of chronic diseases such as epilepsy has long been underestimated.

As the world’s fourth common neurological disorder, epilepsy has affected over 65 million people worldwide and has a prevalence of 7.15‰ in China, which means that nearly 1 out of 100 people, no matter within or out of an epidemic area, suffers from epilepsy [5]. More importantly, cases of epilepsy are not evenly distributed in the world, as a significantly higher portion of patients with epilepsy (PWEs) live in resource-limited regions [6, 7], places that are also vulnerable to epidemic outbreaks, thus bringing a new problem as to how to manage epilepsy during an epidemic.

Similar to many other chronic diseases, epilepsy management requires regular follow-up and sustainable medicine supply [7]. However, these medical resources are difficult to obtain during an epidemic. On the other hand, successful control of epidemics such as COVID-19 epidemic demands the cut-off of routes of the possible transmission [8]. In such case, controlling the flow of population as well as community containment would be inevitable, which may affect medical interactivity between neurologists and PWEs, restrict patient access to medical resources, and make the management of epilepsy more difficult than ever.

To address these challenges, some approaches must be considered, including expanding self-management, using smartphone application-based communications and maximizing the availability of anti-epileptic drugs (AEDs) by all means. Above all, a systematic epilepsy management framework is critical for dynamically responding to epidemic changes. Unfortunately, current epilepsy management framework is generally a periodic outpatient assessment model [9], where a certain medical center is the node for almost all medical services and patients actively transport themselves from home to hospital periodically. Due to the uncertainty of patient flow and unbalanced accessibility of medical resources, such system is less adaptable and cannot cooperate with disease control policies in epidemic areas. Furthermore, the current framework causes a huge waste of medical resources in that the preference of both doctors and patients, rather than objective need, acts as a major driving force for medical service [10]. Hence, a new framework that avoids unnecessary transportation while maximizing the availability of medical resources according to demand is needed. Here we review approaches that help control epilepsy during an epidemic event and raise a new conceptual management framework.

## Self-management of epilepsy

Although not so efficient as many caregivers expect, self-management has its value for chronic diseases [11]. Self-management is characterized by abilities of patients to detect and manage their own conditions. These abilities include management of both medical and non-medical issues such as emotion or role changing [12]. Luedke et al. summarized six aspects of self-management for PWEs based on a systematic review, which are knowledge acquisition, problem solving, medication management, health behavior changing, symptom monitoring, and safety promotion [13]. PWEs have educational needs on two major classes of information for self-management. The first is predesigned knowledge on epilepsy and related issues while the second being a practical guide to self-management of other conditions, especially the psychosocial therapy, and applying it in PWEs [13]. The predesigned epilepsy-specific program largely involves basic knowledge on epilepsy, medication management, and problem-solving suggestions, while psychosocial therapy mainly focuses on health behaviors and related knowledge. To be noted, the details of self-management differ among studies [14–16], making it difficult to evaluate the efficacy. A potential solution to this problem is to establish a network that integrates different programs and evaluate their efficacy, effectiveness and dissemination [17].

However, during an epidemic, the content of these programs requires upgrading. Information on the epidemic should be incorporated into the knowledge education and health behavior changing programs. Sufficient information on transmission routes and prevention approaches of epidemics is critical for epidemic control [8].

Strong mental and emotional health support is another aspect of importance [18] due to the fact that an estimate of 30–50% of PWEs suffer from anxiety even without an epidemic [19–21]. On the other hand, epidemic itself can raise public stress. One example is that during the SARS outbreak in 2003, the suicide rate in Hong Kong increased significantly due to loneliness and disconnectedness [22, 23]. More recent data during the COVID-19 outbreak showed that 53.8% of people suffer moderate to severe stress from epidemic and 28.8% to 44.7% develop anxiety [24, 25]. Behavioral intervention taught through the self-management program has been proven beneficial for anxiety in both epilepsy [26] and epidemic [8, 27].

Approaches to deliver self-management knowledge can also be influenced by the epidemic. Face-to-face training, including group design, is the major method of teaching for PWE self-management, which is usually conducted for 4 to 41 h [13]. However, it is unclear during an epidemic that whether these trainings are still available and to what extent can they reach PWEs. Another concern is that bringing patients together to a teaching class could increase the risk of infection. An alternative method is to group and train them through internet [28], although it would be hard to cover elder people [29].

The advantages of self-management during an epidemic could be more than epilepsy control only. The self-management program and PWEs can form a network of information exchange, which may be helpful in the control of epidemic. Such network, especially that online [30], provides a direct route for caregivers to contact PWEs, thus paving the way for gathering self-reported information on health and other epidemic-related information. Furthermore, PWEs in the system are trained to cope with epilepsy-related issues, thus avoiding repeated visits to the hospital, lowering the risk of exposure to the epidemic. Even under circumstances that require hospital support, the problem-solving training can help perform reasonable decision-making and avoid unnecessary blindness in seeking for medical help.

However, self-management has limits. Studies have suggested that the efficacy of self-management from different programs varies significantly [31]. Some studies [14] even showed that group education is less effective in improving the quality of PWE’s life. Patients in the program should be aware of the side-effects of over-reliance on self-management and exaggeration of its role in medical system [13]. They must understand that they still in many ways require professional medical support, including hospitalization. Several concise thresholds for seeking medical help must be emphasized in order to keep PWEs safe. It is also NOT recommended in any condition to self-manage epidemic at any time!

## Smartphone application and remote medical care in epilepsy management

Smartphone applications (APPs) and other remote medical care approaches are familiar things to patients with chronic disease. In epilepsy alone, a series of studies [32–34] have tested the value of Apps in management of seizures and other issues. Digital technology in the management of epilepsy features a new trend in epilepsy control. There has been a rapid increase of available Apps for PWEs. From the end of 2008 to 2013, the number of epilepsy-related Apps increased from ground zero to 28 [34]. Additionally, an overwhelming majority of these Apps are free for use. In 2018, Escoffery et al. reviewed over 20 Apps from Apple Store for their functionality, esthetics, and information contained. They found that most Apps in epilepsy management are designed for adults and focused on treatment, seizure tracking, response and safety, which cover a significant portion of self-management [32]. Although the population sampled was small, Apps for epilepsy management significantly improved users’ epilepsy knowledge, personal safety management and adherence to AEDs [35, 36].

Apps are considered as a new tool for the self-management program for delivering information and the potentials of Apps on PWE management may be underestimated. However, many new attempts have been made to develop more efficient epilepsy-related Apps. For example, to raise social awareness of epilepsy and meet the social needs of PWEs, an App has been developed to offer social network function for PWEs. Other think-out-of-box attemps include a specifically-designed game for Vegus Nerve Stimulation (VNS) education [32]. In an exciting study aimed to facilitate diagnosis of epilepsy in community by non-professional local caregivers using an App, researchers revealed a roughly equal specificity for correct diagnosis to physicians [37]. These new attempts expanded the traditional concept of epilepsy control and fulfilled different levels of needs for PWEs, even for caregivers.

During an epidemic, the Apps stand out for the convenience of communication, thus significantly reducing unnecessary transportation in seeking for medical assistance while providing necessary information in dealing with epilepsy and epidemic. However, surprisingly, the function of making appointment with or consulting a doctor only exists in two out of 20 Apps in Escoffery’s study [32], suggesting that most epilepsy Apps are unable to provide sufficient medical support to PWE. Meanwhile, Apps with these important functions are designed either by hospitals or health organizations, indicating a necessity for strong collaboration between doctors and program developers. In addition, a recent study in West China found that although only 3.1% of caregivers were using Apps for management of epilepsy, 70.2% of them were willing to do so [38], suggesting a promising future of App-based management.

To fully control an epidemic, additional functions of Apps are needed. Since information gathering from individuals during an epidemic is critical, self-reporting of personal well being is promising. Self-reporting has been widely practiced in China to control COVID-19 outbreak. In China, a health QR code will be generated once a user completes self–report of his (or her) health through an App in the smart phone, which grants the access to train, subway, airline and other public places. However, these reports are not compulsory due to ethical considerations and must be gathered with consensus, limiting its reliability. Transparency of official information on the distribution and number of infected patients in certain areas also helps control epidemics [8, 39, 40]. During the COVID-19 epidemic in China, Apps such as Alipay or Baidu provide this official information on their pages. They post up-to-date information through data-mining and list distributions of confirmed patients according to areas. In addition to digital mapping, these Apps even provide the exact buildings in which patients were discovered. Although this may raise ethical concern, people can be guided to keep a distance from epidemic areas. To be noted, these Apps are not specifically designed for epidemic, but rather flexible in their functions due to modular design. They may provide reference for epilepsy management Apps to meet different needs.

Apps aiming at improving mental and emotional health are also important. Mental health services are nowadays largely available online, even during epidemics [24], facilitating the route for intervention. Hence, the proposed new approaches for treatment such as structured letter therapy [41] for consultation on mental problem during COVID-19 epidemic can be easily deployed in App. These Apps are largely available online and have helped different groups of patients improving their mental and emotional health. In a review, 27 Apps on mental health were evaluated for their functions [42], suggesting that mental health Apps with cognitive behavior treatment targeting depression and anxiety are available. These Apps also included self-reporting function which not only keeps caregivers informed about patient’s mental status, but also has great potentials in sharing epidemic information. Apps with integrated epidemic control, mental health and epilepsy management functions are feasible and applicable.

However, there are several controversial issues on Apps. First is the acceptance rate of Apps among PWEs. A study performed in 2015 [43] found that 58.23% of mobile-phone users had tried at least 1 health App. Also, people who are more likely to use health Apps are those younger, more educated and with higher income. Interestingly, a more recent survey found that those with good or excellent health condition turned out to be the largest user group for health Apps [44]. For PWEs, according to Liu’s study [45], 66.7% of surveyed PWEs considered Apps in epilepsy beneficial and 65.5% would like to accept using free Apps. Again, young people and urban PWEs are more likely to use Apps. Another interesting finding of this study is that the attitude of patients with poor seizure control or having adherence issues turned out to be more positive towards Apps. Overall, these data indicated that health Apps have a promising acceptance rate in the general population, but their use may still be refused or failed in the elder population and those who live in resource-limited areas.

The second problem is withdrawl from Apps. In Krebs’s study [43], up to 45.7% of those who ever used health Apps had stopped using some of these Apps. The primary reasons are high data entry burden, hidden cost as well as degenerating interest [43]. This tells again for developers that the quality of programming, content and visualization is important for a successful App. Moreover, as we demonstrated above, the lack of direct communication with doctors as well as the lack of many other appealing functions may be potentially relevant to the withdrawal. Studies must be done to further understand the core need from PWE before designing an App.

## Availability of AEDs

Although new therapies are emerging continuously, AEDs still are the very bases of epilepsy treatment [46]. There are currently two major commercial ways for PWEs to obtain AEDs: getting medicine in hospital and buying AEDs from pharmacy. Theoretically, PWEs can access AEDs according to their preference. However, practically, there are some factors influencing their buying decision, including price, distance from home, insurance coverage, brand and, above all, availability of AEDs.

Availability of AEDs is a major concern of epilepsy management, especially in resource-limited areas. A study in Zambia carried out in 2010 found that 49.1% of pharmacies did not even have AEDs and only 4 kinds of first-generation AEDs were available [47]. The same situation also occurs in other middle- and low-income countries. A study in 2012 reported that the available rate of basic AEDs such as phenytoin, carbamazepine, valproic acid and phenobarbital is less than 50% in 46 countries [48]. What makes the matter worse is that these developing countries are also susceptible to epidemics, which can further deteriorate the availability of AEDs, if not all medical resources. During the 2003 SARS outbreak, even in major cities, there was a shortage of certain medicines [39], suggesting that the hospital- and pharmacy-centered supply of medicine has limits, and could not adapt to changes during an epidemic.

Another problem of this system is the requirement of active transportation of patients: in order to obtain necessary AEDs, PWEs have to go to pharmacies or hospitals regularly. Such frequent flow of patients is unfavorable for epidemic control and may further aggravate the situation due to the lack of medicine. Keeping daily supply of AEDs for hospitals and pharmacies could also be challenging. Particularly, transporting medical resource into quarantine areas during an epidemic is difficult. In addition, the focus at such time is always on gathering resources to control the infectious disease, threatening the supplement priority of AEDs.

However, there have been some new ways to solve this paradox. Online pharmacy with non-contact express delivery service is a promising option. One such example is the major online shopping websites taobao.com and jd.com in China. Both sites sell AEDs as online pharmacies with permission. AEDs including carbamazepine, valproic acid, phenytoin, lamotrigine, levetiracetam, topiramate, oxcarbazepine, gabapentin pregabalin, lacosamide and perampanel are all available online, except phenobarbital, which is considered a psychotropic medicine requesting special prescript from professionals. Moreover, these AEDs could be sent to any street address (except those in compete quarantine) in any county by express.

There are some potential risks for such online shopping during epidemic. First is the concern that express may cause spread of disease, especially by the final door-to-door dispatching. During the COVID-19 epidemic, companies in China established a way called ‘non-contact approach’ to deal with this concern. To be brief, the delivering system consists of two parts: intelligent express cabinet placed in the community and certain sets of protective equipment for couriers. In this non-contact system, all packages are delivered to the cabinet instead of to the door. The customer will receive an encrypted code corresponding to a cell of the cabinet where the package is stored, thus preventing direct contact with couriers. On the other hand, couriers are provided with masks, gloves, and glasses in order to prevent direct contact with contaminated goods. The efficiency of such system has been proven in recent COVID-19 epidemic and express companies worldwide have referred to this approach.

The second concern for online AED shopping is the time cost for shipping, which may be further prolonged during an epidemic. A reasonable solution is to post the estimated time for shipping on the buying page so that the PWE could know roughly the time for shipment. Another solution is to establish a ranking system for express companies that provide expedited shipping for medicines. However, such ideas might not come into reality without the support of governmental policy, since these acts will inevitably raise the cost for express companies.

A combination of local and online pharmacies could further solve this problem. Local pharmacy has the advantage of sustainable source of AEDs, while the websites could provide information on where AEDs are available in local pharmacy, offering reserving services to further complete local availability. Express to certain pharmacy is easier and faster to achieve, and also is an alternative way for non-contact express service, where pharmacy itself becomes an intelligent express cabinet. Still, systematical reforms of both pharmacies and the government are needed to maximize efficiency and balance the operating cost. However, under the epidemic situation, it is highly recommended that PWE keep sufficient storage of AEDs at all time and be readily resupplied with AEDs through all available sources.

In resource-limited areas where AED availability is extremely limited, strategies described above are difficult to carry out. The convulsive epilepsy management program in rural West of China may offer an answer to this crisis [49]. The program enrolled any patient with convulsive epilepsy through a door-to-door interview conducted by local Central Disease Control and primary care physicians, fully covering the population in the program area. The patients received phenobarbital as initial treatment and were followed up monthly for efficacy and safety. Phenobarbital in the program was purchased directly from the pharmaceutical company by program fund, which thus ensured the long-term availability. Although PWEs in this program had limited options of AEDs, convulsive epilepsy was successfully managed in over 7 000 PWEs with convulsive seizures with acceptable adverse events. During the COVID-19 epidemic in 2020, there was no report of AED insufficiency in any of the program areas. This suggests that official act, rather than dependence on local resources, is applicable for management of epilepsy during an epidemic in resource-limited areas.

## Proposed epilepsy management framework under epidemic situations

**Fig. 1:**
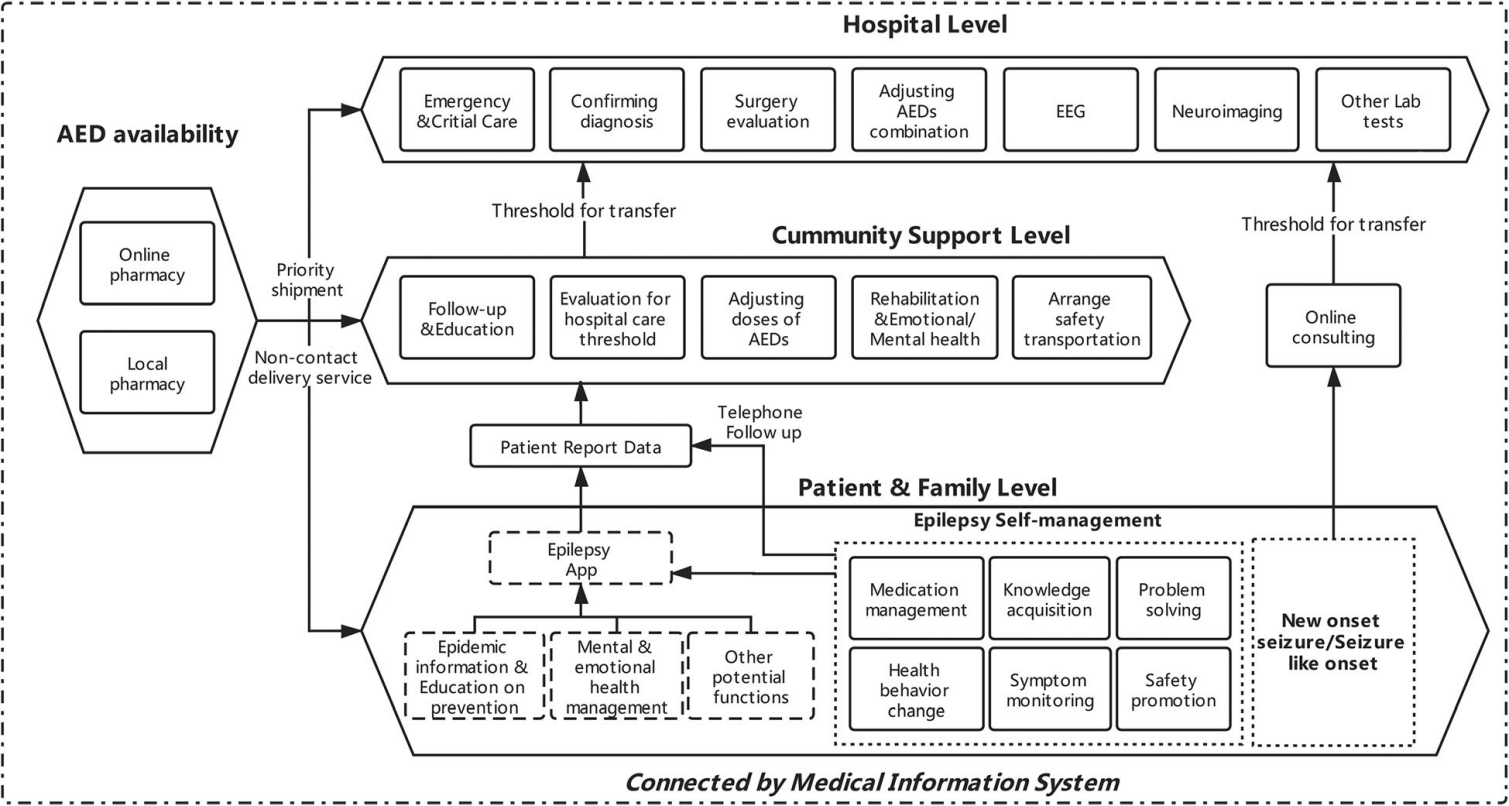
Schematic diagram of the three-level management framework for epilepsy control during an epidemic

There are limited studies on different management frameworks under different conditions, and only one framework was specifically designed for epilepsy during an epidemic [50]. Here we establish a new three-level framework for epilepsy management during an epidemic according to literature and based on our experience (Fig. 1). Generally, different levels refer to different institutions or medical entities with unequal capability of epilepsy treatment. Added to these levels is the AEDs availability and delivery system, both online and local, which determines what kind of treatment could be used during an epidemic. The whole framework is boxed by a secured medical information system which intends to minimize the unnecessary flow of patients while maximizing the continuous communication among different compartments.

These three levels, namely, patient & family level, community support level and hospital level, are geographically independent, making quarantine of certain level possible during an epidemic. Also, they conceptually represent different management measures in epilepsy. The patient & family level focuses on self-management, including all six components mentioned above [32] and is facilitated by epilepsy-related Apps, while the community support level, consisting of general physicians and other local caregivers from the community, acts both as a threshold for hospitalization and an outpost for providing basic intervention, including education, adjusting AED doses, rehabilitation and mental health management. By emphasizing the role of the community, this system requires patient to report their health problems to community supporters for evaluation before going to hospital. The top hospital-level provides medical support for patients with new-onset epilepsy and those in urgent needs.

### New-onset epilepsy

Normally, patients with first seizure-like onset, either provoked or unprovoked [51], should be sent to hospital for systematic examination if possible. However, such active response to epilepsy as a chronic disease during an epidemic is debatable. Since a clear medical history is key to the diagnosis, online consulting with community caregivers or even epileptologists is recommended before directly visiting a hospital. This approach could avoid blind seeking for medical support and ease the psychological impact from first seizure. Considering that neuroimaging and electroencephalogram are both necessary for diagnosis of new-onset epilepsy, it is important to make an appointment for these tests during an epidemic. Patients must be informed the importance of these examinations for determining the etiology and diagnosis. However, they should also understand that (a) a number of patients will not suffer second seizure in a relatively long period of time, which will even not recur at all [52]; (b) if the medical history is typical, the diagnosis of epilepsy can be made without these tests [53]; (c) some provoked seizures (i.e. alcohol-induced seizures) can be prevented by avoiding triggering factors [51]; (d) even with neuroimaging and electroencephalogram tools, the etiology of some onsets may be still controversial due to many factors. Since online prescription systems have already been in legal use, a further decision as whether to start AEDs immediately can also be discussed during online consulting.

On the other hand, in the presence of any clue of the following situations, hospitalization should be recommended (thresholds of transfer to hospital for patients with first onset) (Any of below):

1. Evidence of prolonged seizures which last for more than 5 min
2. Seizures are accompanied by other symptoms, including developmental problems, impaired cognitive function, paralysis, psychological syndrome, and other neurological problems that cannot be explained by seizure itself
3. Recurrent seizures that cannot be explained by a specific cause or triggering factor
4. Patient has a clear family history of epilepsy
5. Atypical onset that could not distinguish seizures from syncope
6. Elder patient (> 60 years)

Under such scenarios, community support is needed to secure a safe transfer route to the hospital in order to lower the risk of epidemic infection.

For the ongoing pandemic, there is no evidence that COVID-19 could directly induce epilepsy [54]. A recent study in Wuhan identified only one seizure in a severe case [55]. Another report from Japan described a COVID-19 related meningitis/encephalitis case presenting with seizures [56]. However, in this case, the RNA test was only partially positive in cerebral spinal fluid and was negative on nasopharyngeal swabs sample.

### Follow up

Follow up during an epidemic is achieved through a medical information system consisting of two parts. The first is a unified Smartphone App for patients with daily reminder for medicine adherence, seizure recording, a patient outcome-reporting system and an online communication function. It works not only as a tool for self-management, but also as a node for epidemic education, emotional intervention and other potentials, such as social function. All information gathered from App will be presented regularly (i.e. every 1 month) as patient-reported outcomes for evaluation by community caregivers. The second part targets those who do not have the ability or are unwilling to use Apps. Regular follow-up through telephone by community is recommended. Two sets of thresholds are set for community caregivers to screen for patients requiring further interventions:

First set: requesting community medical intervention (primary physicians & caregivers) (Any of below):

1. Efficacy Issue (The patient did not reach seizure free in the last month)
2. Adherence Issue (The patient failed to understand protocol or had more than 1 protocol-violence in the last follow-up period)
3. Adverse Events (Any event that has mild or moderate influence on patient’s daily activity)
4. Emotional Instability (Patient presented with mild to moderate anxiety or depression symptoms, or obvious stress in response to the epidemic)
5. Information or Rehabilitation Needs

Second set: requesting intervention by hospital and transfer to hospital (Any of below):

1. Patient presented with status epilepticus (defined as any tonic-clonic seizure lasting for more than 5 min, focal or other types of seizure for more than 10 min, or patient did not recover between two seizures [57])
2. Serious Adverse Events (Events that significantly influence patient’s daily activity, or require additional medicine, or require inpatient treatment)
3. Lack of Efficacy (A reduction of seizure frequency in the last month of less than 50% of baseline or any deterioration of seizures, either in frequency or in manifestation)
4. New Psychological Manifestations and Severe Emotional Impact (severe anxiety or depression)
5. Comorbid with Other Diseases that Require Hospitalization

By reviewing patient-reported data, such system allows community caregivers to filter and separate patients into different subgroups according to their actual medical demand. Patients below such threshold would maintain their current management and stay within the first level. Those beyond the first threshold need to contact, either in an online or in a face-to-face manner, the community caregivers to acquire appropriate intervention. Since the community-based medical care is within a certain geographical range and patients have no necessity to blindly seek for medical care, this framework can theoretically minimize the mobility of patients, thus contributing to epidemic management as well. However, if a patient reaches the second threshold, he (or she) would be in need of hospital care. A transport arranged by community is recommended to secure a safe and swift pathway.

Another important part of this framework is the AED availability, which stands beside the three levels. As discussed above, multiple measures are taken to achieve high availability of AEDs, including maintaining storage for local pharmacy, online reservation of AEDs, priority shipment and non-contact express delivery service. Finally, Fodjo’s [50] practice as well as our own experience [49] in rural West of China suggests that strong governmental act is needed for successful management of both epidemics and epilepsy, especially in resource-limited areas. Practical test is needed for further refinement of this framework.

## Conclusion

The management of epilepsy as well as many other chronic diseases under an epidemic is largely unknown. Emerging approaches such as self-management, Apps and non-contact delivery service are promising solutions to this problem and can be integrated into future management frameworks. Meanwhile, a joint effort from health providers, society and government is essential for addressing the challenges.

## Data Availability

Not applicable.

## Abbreviations

COVID-19: Corona virus disease 2019
SARS: Severe acute respiratory syndrome
PWE: Patient with epilepsy
AEDs: Anti-epileptic drugs
Apps: Applications
VNS: Vegus Nerve Stimulation
